# Native Arbuscular Mycorrhizal Fungi Promote *Plukenetia volubilis* Growth and Decrease the Infection Levels of *Meloidogyne incognita*

**DOI:** 10.3390/jof10070451

**Published:** 2024-06-27

**Authors:** Mike Anderson Corazon-Guivin, Sofía Rengifo del Aguila, Ronan Xavier Corrêa, Deyvis Cordova-Sinarahua, Leonor Costa Maia, Danielle Karla Alves da Silva, Gladstone Alves da Silva, Álvaro López-García, Danny Coyne, Fritz Oehl

**Affiliations:** 1 Laboratorio de Biología y Genética Molecular, Universidad Nacional de San Martín, Jr. Amorarca N° 315, Morales 22201, Peru; sofyren@hotmail.com; 2 Center of Biotechnology and Genetics, Department of Biological Sciences, Universidade Estadual de Santa Cruz, Rodovia Jorge Amado Km 16, Ilheus 45662-900, Brazil; ronanxc@uesc.br (R.X.C.); dcsinarahua.ppgpv@uesc.br (D.C.-S.); 3 Departamento de Micologia, Centro de Biociências, Universidade Federal de Pernambuco, Av. da Engenharia s/n, Recife 50740-600, Brazil; leonorcmaia@gmail.com (L.C.M.); daniellekarlas@yahoo.com.br (D.K.A.d.S.); gladstone.asilva@ufpe.br (G.A.d.S.); 4 Departamento de Microbiología del Suelo y la Planta, Estación Experimental del Zaidín (EEZ), CSIC, 18008 Granada, Spain; alvaro.lopez@eez.csic.es; 5 International Institute of Tropical Agriculture (IITA), Ibadan 200113, Nigeria; d.coyne@cgiar.org; 6 Agroscope, Competence Division for Plants and Plant Products, Plant Protection Products-Impact and Assessment, Müller-Thurgau-Strasse 29, 8820 Wädenswil, Switzerland; fritz.oehl@agroscope.admin.ch

**Keywords:** biofertilizer, bioprotectant, mycorrhizal symbiosis, *Nanoglomus plukenetiae*, *Rhizoglomus variabile*, root knot nematode

## Abstract

The use of arbuscular mycorrhizal fungi (AMF) offers promising benefits to agriculture in the Amazon regions, where soils are characteristically acidic and nutrient-poor. The purpose of this research was to investigate the potential effects of two recently described species of AMF (*Nanoglomus plukenetiae* and *Rhizoglomus variabile*) native to the Peruvian Amazon for improving the plant growth of *Plukenetia volubilis* (inka nut or sacha inchi) and protecting the roots against soil pathogens. Two assays were simultaneously conducted under greenhouse conditions in Peru. The first focused on evaluating the biofertilizer effect of AMF inoculation, while the second examined the bioprotective effect against the root knot nematode, *Meloidogyne incognita*. Overall, the results showed that AMF inoculation of *P. volubilis* seedlings positively improved their development, particularly their biomass, height, and the leaf nutrient contents. When seedlings were exposed to *M. incognita*, plant growth was also noticeably higher for AMF-inoculated plants than those without AMF inoculation. Nematode reproduction was significantly suppressed by the presence of AMF, in particular *R. variabile*, and especially when inoculated prior to nematode exposure. The dual AMF inoculation did not necessarily lead to improved crop growth but notably improved P and K leaf contents. The findings provide strong justification for the development of products based on AMF as agro-inputs to catalyze nutrient use and uptake and protect crops against pests and diseases, especially those that are locally adapted to local crops and cropping conditions.

## 1. Introduction

Food security is a significant global challenge that aims to ensure availability, access, use, and stability to satisfy the fundamental human right to food [1]. Due to a rapidly rising global population, there is an ever-rising demand for food associated with the increasing number of people to feed. There is consequently a continuous need to intensify and adapt agricultural practices to increase productivity [2]. However, such intensification of agricultural practices cannot be sustained without increasing the use of synthetic inputs, which has ecological as well as human safety issues associated with it [3,4]. In addition to the strive to improve productivity, therefore, there is also the necessity to achieve sustainable and ecologically equitable production through the identification of alternatives that can improve resource use efficiency.

Arbuscular mycorrhizal fungi (AMF) facilitate the long-term benefits of soil fertility, plant nutrition, and protection against pathogens, as well as maintaining agroecosystem services and dynamics with a reduction of environmental degradation [4]. They establish an obligate symbiotic association with the roots of ~80% of terrestrial plant species, in which the plant supplies carbon and lipids to the fungi and the fungi deliver water and nutrients to the plant [5,6]. For agriculture, they offer a promising, largely untapped potential towards more sustainable agriculture. They assist plant acquisition of nutrients for improved growth but also, by eliciting host resistance to soil-borne pests and diseases, result in greater shoot growth [7].

*Plukenetia volubilis*, called sacha inchi in Spanish and inka nut in English, produces eatable, delicious seeds. It is a traditional Amazonian crop that dates back millennia. Lately, it has received increasing attention due to its remarkable seed nutritional contents, which include omega fatty acids (ω3, ω6, and ω9) [8], proteins (22–30%), vitamin E (tocopherols and tocotrienols), and natural antioxidants, and its associated benefits for human health, including benefits for nutritional and pharmaceutical purposes [9,10]. In Peru, national production reaches 2785 tons, with the San Martín region being the main producer, contributing 1797 tons, which represents 64.5% of the country’s total production [11]. It is cultivated in combination with various other plants as live stakes and increasingly under more intensive conditions in monoculture. Additionally, it grows across a broad range of soils and altitudes. For example, it can be found at altitudes ranging up to 1490 m above sea level (m.a.s.l.) [12]. However, *P. volubilis* appears to be highly affected by a particularly damaging and fatal root disease complex caused by root knot nematodes (*Meloidogyne* spp.) and fungal wilt pathogens (*Fusarium* spp.) [13].

Studies assessing the application of AMF to *P. volubilis* are scarce. However, recent research by Wiriya et al. [14] demonstrated that inoculation with *Acaulospora* sp. generally improved plant growth and development. Additionally, Tian et al. [15] found that inoculation with *Glomus versiforme* and *Paraglomus occultum* enhanced the crop’s ability to withstand drought conditions. Given the recent descriptions of two native species of AMF, *Rhizoglomus variabile* and *Nanoglomus plukenetiae* [16,17], and that *P. volubilis* is indigenous to the Amazonia region, the current study was established to investigate the potential of these indigenous AMF to offset the impact of the *Meloidogyne* spp. and additionally improve the productivity of this interesting crop. This study aims to evaluate the biofertilizer and bioprotective potential of single and combined inoculations of two native AMF species for seedling development and early plant growth of *P. volubilis* plants. We hypothesized that inoculating *P. volubilis* plants with *R. variabile* and *N. plukenetiae* alone or in combination will enhance the development of morphological traits and contribute to a level of tolerance against *M. incognita* infection.

## 2. Materials and Methods

### 2.1. Plukenetia Volubilis Seed

**Figure 1:**
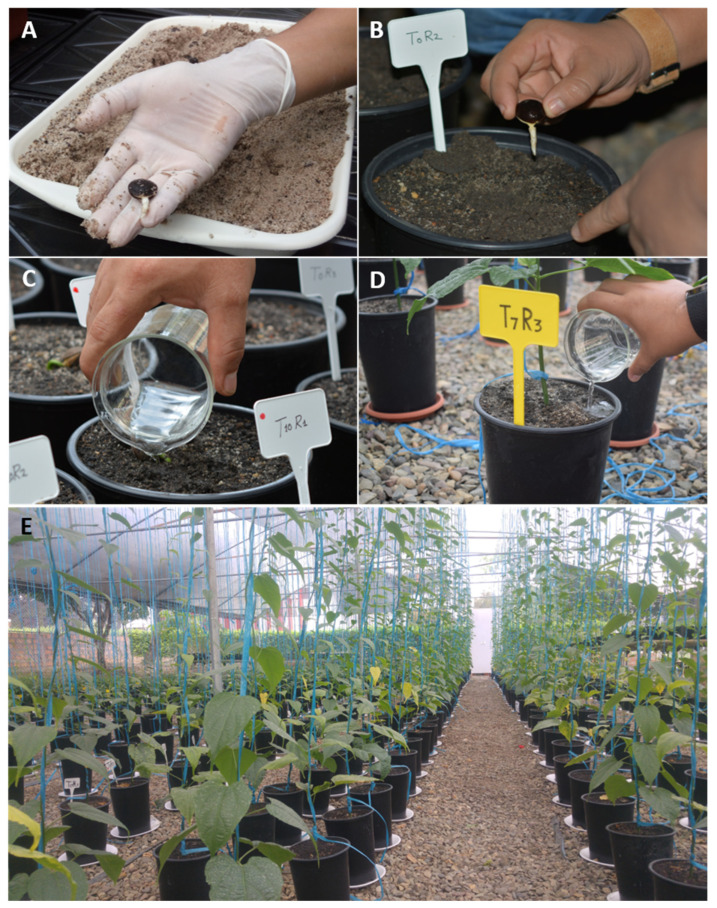
Inoculation of *Plukenetia volubilis* seedlings with arbuscular mycorrhizal fungi (AMF) and *Meloidogyne incognita*. (**A**) Germination of *P. volubilis* seeds in sterile sand. (**B**) Sowing of pre-germinated *P. volubilis* seeds on substrate with AMF. (**C**) Inoculation of *P. volubilis* seedlings with *M. incognita* (0 days). (**D**) Inoculation of *P. volubilis* seedlings with *M. incognita* 45 days after sowing. (**E**) Experimental layout of *P. volubilis* seedlings in the greenhouse 60 days after sowing.

Ripe capsules (fruits) of *P. volubilis* (ecotype Shanantina) were collected from healthy plants without signs of pest or diseases in 2018 from Lamas province, San Martín department, Peru (06°26′47.3″ S 076°31′44.00″ W; 382 m a.s.l.). Viable seeds with similar color (dark brown), size (1.5–2.0 cm), and shape (ovoid and bulging seed) were selected according to Guerrero-Abad et al. [13]. Seeds were surface-sterilized by immersing in 0.05% sodium hypochlorite for 2 min and 95% ethanol for 2 min and then rinsed in sterile distilled water three times. The seeds were vertically positioned with the hilum directed downwards in a tray [13], covered with a thin layer of sieved coarse sterile sand (1 cm thick), and irrigated daily with water during the first week until pre-germination (Figure 1A).

### 2.2. Mycorrhizal Inoculum

Pure cultures of the isolates *R. variabile* and *N. plukenetiae* were obtained from the collection of Arbuscular Mycorrhizal Fungi of the Laboratorio de Biología y Genética Molecular of the Universidad Nacional de San Martín (Peru). Our previous experiments show that *R. variabile* and *N. plukenetiae* have a high potential for biofertilizer and growth promotion in coffee (*Coffea arabica*) seedlings [18]. These isolated AMF were multiplied using *Sorghum vulgare*, *Urochloa brizantha,* and *Medicago sativa* together over several continuous cycles in the greenhouse (06°35′28″ S, 76°18′47″ W) under the environmental conditions as described by Corazon-Guivin et al. [19]. Information about morphological and molecular characterization, including DNA extraction, PCR, cloning, sequencing, and phylogenetic analyses of *R. variabile* and *N. plukenetiae,* is available in Song et al. [17] and Corazon-Guivin et al. [16]. For single inoculation assays, an inoculum of each isolated AMF (Rv: *R. variabile* and Np: *N. plukenetiae*) was delivered using 20 g of substrate, which contains segments of mycorrhizal roots, hyphae, and ~1500 AMF spores. For dual inoculation assays (Rv + Np), 10 g of each inocula was combined to inoculate 20 g per inoculated pot.

### 2.3. Inoculation of Plukenetia volubilis Seedlings

Each inoculum (20 g of Rv, Np, and Rv + Np) was mixed with 3 kg of previously sterilized substrate (121 °C, 15 p.s.i., 60 min per day/three consecutive days) composed of a mixture of field soil and coarse river sand (2:1, *v*/*v*). The textural classification of this substrate was a sandy loam, with pH 4.82, 0.35 dSm−1 electrical conductivity, 1.66% organic matter, 6.5 mg of P kg^−1^, and 63 mg of K kg^−1^ (0.14 K + meq/100 g). Uniform pre-germinated seeds (~1 cm root) were transplanted singularly into plastic 3 L pots filled with 3 kg of sterile substrate containing AMF inoculum (Figure 1B).

### 2.4. Experiment #1: Impact of AMF Inoculation on Plukenetia volubilis Growth and Physiology 

#### 2.4.1. Experimental Design

The experiment was performed from May to July 2018 and comprised 4 treatments, each with 12 replications, arranged in a completely randomized design totaling 48 experimental units (i.e., pots, Table 1) in the greenhouse at Universidad Nacional de San Martín, Tarapoto (06°35′28″ S, 76°18′47″ W). Four experimental treatments included a non-mycorrhizal control and single and dual inoculation of *R. variabile* and *N. plukenetiae*. Each pot contained one *P. volubilis* seedling cultivated for 75 days in the greenhouse and watered to field capacity at 3-day intervals until completion. 

#### 2.4.2. *Plukenetia volubilis* Growth, Physiology, and Mycorrhizal Characterization

Plant height (cm), stem diameter (mm), and number of leaves growth parameters were recorded at 10-day intervals over 75 days from the 15th day. At experiment completion, total fresh biomass (g), dry biomass (g), chlorophyll content (SPDA, Minolta Camera Co., Ltd., Osaka, Japan), leaf area (cm^2^, ImageJ FIJI) and leaf nitrogen (N, mg kg^−1^), phosphorus (P, mg kg^−1^), and potassium (K, mg kg^−1^) content were determined. The leaf N concentration was obtained using the Kjeldahl method [20], the leaf P concentration was obtained through digestion in HNO_3_:HClO_4_ (4:1) and spectrophotometry in UV-Vis (λ = 420 nm), and the K concentration was obtained through digestion in HNO_3_:HClO_4_ (4:1) and atomic absorption spectrophotometry (Model Varian, AAS Spectra 55B, Victoria, Australia). 

Mycorrhizal root colonization (%) and spore density (per 10 g of soil) were also determined. For this, the roots were rinsed and cut into 1–2 cm fragments. These root fragments were cleared by boiling in 10% (*w*/*v*) KOH and stained with Parker Quink ink in lactoglycerol according to the modified method of Vierheilig et al. [21]. Twenty pieces of roots per plant were observed under an optical microscope at 20× magnification and evaluated according to Brundrett et al. [22]. To assess spore density, they were first isolated using the wet sieving and decantation method [23], followed by sucrose centrifugation. Subsequently, they were quantified using a stereoscopic microscope (at 20× magnification, Eclipse E200, NIKON, Tokyo, Japan).

### 2.5. Experiment #2: Impact of AMF Inoculation on Plukenetia volubilis against Meloidogyne incognita

In the second experiment, AMF inoculations were challenged with nematodes. The inoculation of AMF in *P. volubilis* was the same as in the previous experiment.

#### 2.5.1. Nematode Inoculum

Egg masses of *M. incognita* were individually removed from naturally infected *P. volubilis* root samples collected from plantations located in Aucaloma, Lamas Province, Peru. Each egg mass was placed independently in 5 mL Petri dishes in distilled water [24] and 100–200 freshly hatched infective juveniles (J2) used to inoculate 30 × 15-day-old *P. volubilis* plants in pots filled with sterilized substrate. After 60 days, J2 was extracted from the galled roots according to Hussey and Barker’s [25] method, modified by Atamian et al. [24], and quantified. Information about the molecular characterization of *M. incognita* is available in Guerrero-Abad et al. [13].

#### 2.5.2. Experimental Design

A two-factorial experiment, with 8 treatments and 12 replications per treatment, was conducted using a completely randomized design over 75 days (see Table 2) from May to July 2018. The first factor included timing of *M. incognita* infestation at two levels (0 or 45 days after inoculation of AMF), and the second factor was AMF inoculation with four levels: single AMF isolated (Rv, Np), their combination (Rv + Np), and non-inoculated (Figure 1C,D). Each replicate consisted of a pot with one *P. volubilis* plant, as described in Experiment #1. Nematodes were inoculated using 3000 freshly hatched J2 suspended in 10 mL of distilled water in 4 holes (10 cm deep, 0.5 cm diameter) equispaced around each seedling [13]. All treatments were compared against a non-inoculated control (no AMF, no Mi). 

#### 2.5.3. *Plukenetia volubilis* Growth, Physiology, and Mycorrhizal Characterization and Evaluation of *Meloidogyne*

Plant growth, physiology, and AMF parameters as outlined in Experiment #1 were recorded. In addition, the nematode density (J2) per 100 g of soil, per planta (J2), and the root infestation level according to Bridge and Page [26] at the end of the experiment were recorded. The reproductive factor (RF) was calculated as follows: RF = final population/initial population, where the final population is the number of J2 counted at the end of the experiment (soil and root) and the initial population is the number of inoculated J2 (3000).

### 2.6. Culture Conditions

Both experiments were conducted in the greenhouse of the Laboratorio de Biología y Genética Molecular of the Facultad de Ciencias Agrarias, Universidad Nacional de San Martín, Tarapoto, Peru from May to July 2018. During this period, the temperature was between 21.4 °C and 38.2 °C, whereas the relative humidity was between 47.9% and 73.8% (Figure 1E). Fertilization was applied weekly with 75 mL of the Long Ashton nutrient solution [27], modified to supply 10.25 µg of P mL^−1^ per pot.

### 2.7. Statistical Treatment of Data

Measured variables in the study were evaluated for normality and homogeneity using Shapiro–Wilk [28] and Levene’s [29] tests, respectively. When assumptions were not fulfilled, data were subjected to a log or square root transformation process according to the case analysis of variance (ANOVA) followed by Tukey’s HSD to test for differences among treatments at *p* < 0.05 [30]. When analyzing root colonization data and spore density, the non-inoculated treatment was excluded. The analyses of variance and mean comparison tests were conducted using the transformed data, which were back-transformed to present the original units. All of the data were analyzed using R version 4.0.2 (R Core Team, 2020).

## 3. Results

### 3.1. Experiment #1: Impact of AMF Inoculation on Plukenetia volubilis Growth and Physiological Responses

**Figure 2:**
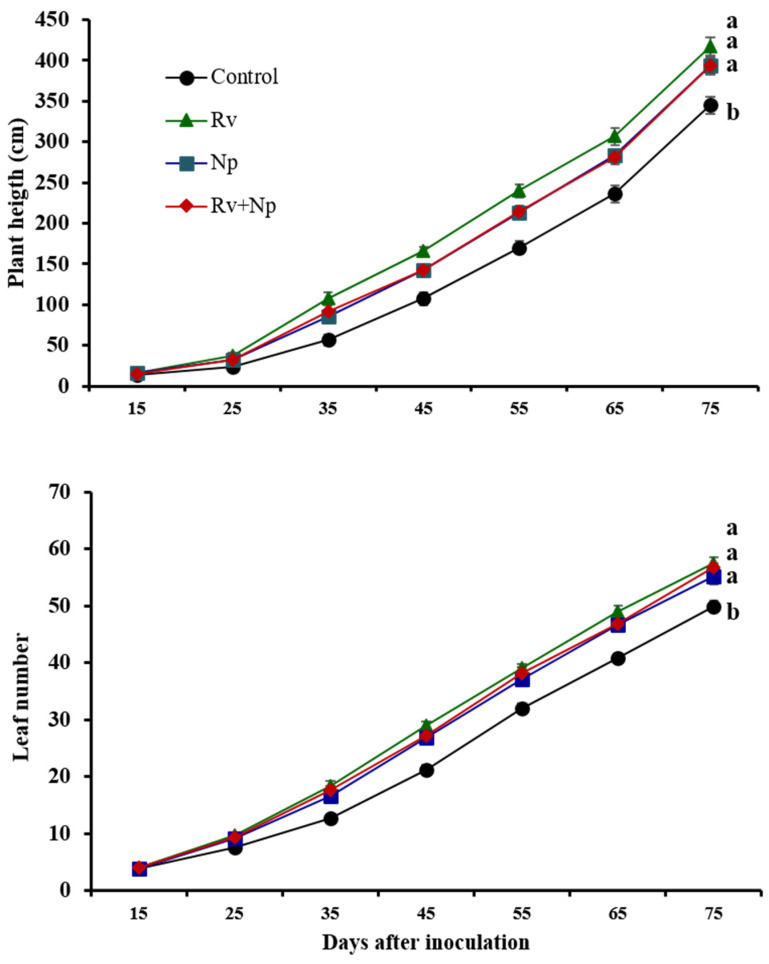
Effect of single and dual arbuscular mycorrhizal fungal inoculation on plant height and leaf number of *Plukenetia volubilis* at 10-day intervals. Means ± standard deviation of 12 replicates. Treatments with the same letter are not significantly different (*p* < 0.05). Control = non-inoculated, Rv = inoculation with *Rhizoglomus variabile*, Np = inoculation with *Nanoglomus plukenetiae*, Rv + Np = inoculation with *R. variabile* + *N. plukenetiae*.

**Figure 3:**
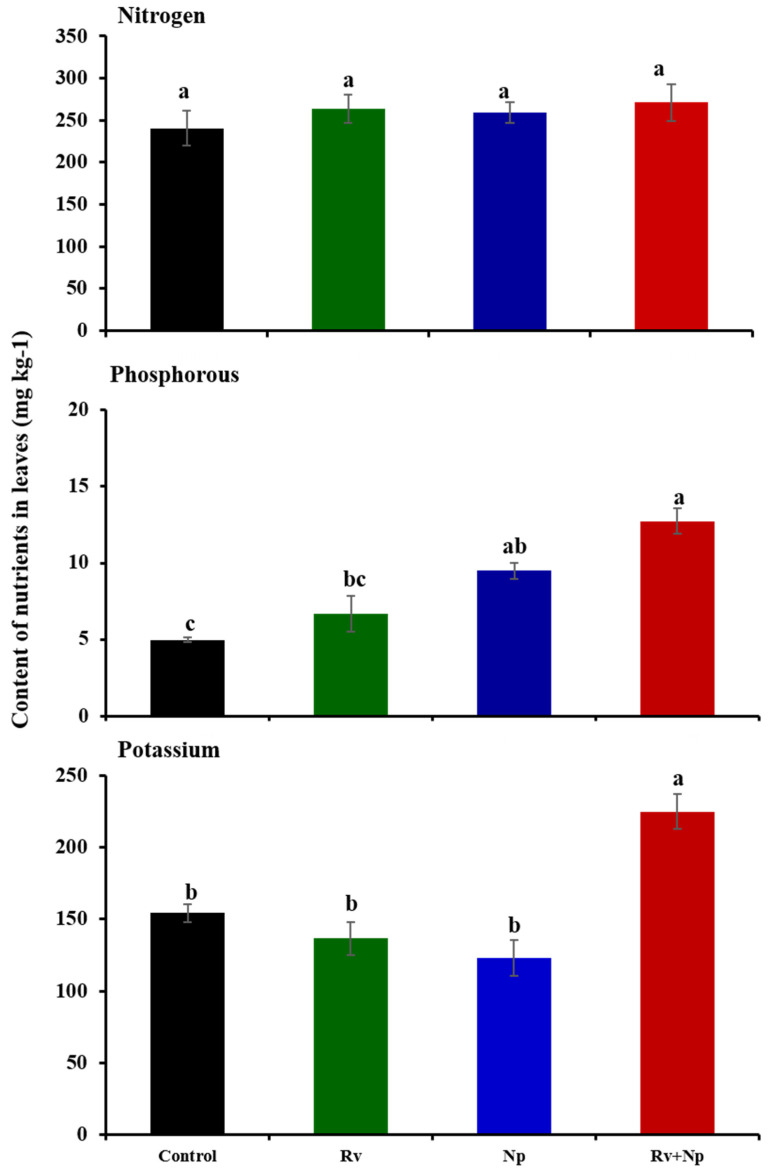
Effect of arbuscular mycorrhizal fungal inoculation on nitrogen, phosphorus, and potassium leaf contents (mg kg^−1^) of *Plukenetia volubilis* after 75 days. Means ± standard deviation of 12 replicates. Treatments with the same letter are not significantly different (*p* < 0.05) Rv = *Rhizoglomus variabile* and Np = *Nanoglomus plukenetiae*.

In general, inoculation with AMF resulted in improved growth and development of *P. volubilis* (Table 3, Figure 2 and Figure 3). Plant shoot weight (total fresh and dry biomass) was up to 1.16 and 1.35 times higher, respectively, for AMF-inoculated plants compared with non-mycorrhizal plants. At 75 days after inoculation, all mycorrhizal plants were significantly taller (1.14–1.20 times, F_3_ = 8.96, *p* < 0.0001) and had more leaves (1.12–1.16 times, F_3_ = 10.46, *p* < 0.0001) than non-mycorrhizal plants (Figure 2). 

Single inoculation with *R. variabile* led to the greatest leaf area (652 cm^2^), which was higher than the leaf area for *N. plukenetiae* and dual-inoculated plants, all of which were 1.32, 1.23, and 1.24 times greater than non-mycorrhizal control plants, respectively. Chlorophyll content (SPAD) was not significantly affected by AMF inoculation. All mycorrhizal treatments demonstrated good AMF colonization of *P. volubilis* roots. The highest root colonization (94.4%) and soil spore density (169 per 10 g of soil) were recorded in plants inoculated with *R. variabile* only, followed by the dual inoculation and single inoculation with *N. plukenetiae* (Table 3). 

Leaf nutrient contents were increased for P (12.7 mg P kg^−1^; F_3_ = 19.20, *p* < 0.0001) and K (224.9 mg K kg^−1^; F_3_ = 17.05, *p* < 0.0001) following dual AMF inoculation, which were 2.5 and 1.5 times higher than non-inoculated plants, respectively. When inoculated individually, *N. plukenetiae* significantly improved P leaf content (1.9 times higher than control plants), but single inoculation treatments did not lead to enhanced K contents, and no AMF treatment affected N content (Figure 3, F_3_ = 0.81, *p* = 0.52).

### 3.2. Experiment #2: Impact of AMF Inoculation on Plant Growth and Meloidogyne incognita Infection

#### 3.2.1. Plant Growth Parameters

Overall, infection by *M. incognita* reduced *P. volubilis* growth and development, which was effectively compensated when inoculated with AMF (Table 4). This recovery against *M. incognita* infection resulted in increased plant growth in mycorrhizal treatments when compared to control plants. Plants generally showed higher growth in treatments infected 45 days after AMF inoculation than those infected on the day of AMF inoculation (day 0). The leaf area, total fresh biomass, and shoot dry biomass were significantly affected by *M. incognita* infection and by inoculation with AMF, while the interaction of both factors was non-significant (Table 4). Leaf area was significantly increased by AMF inoculation regardless of infestation timing with *M. incognita* (up to 1.29 times higher than the control), except for the *N. plukenetiae* and *R. variabile* + *N. plukenetiae* treatments infested with the nematodes at day 0. Dual AMF inoculation induced the highest leaf area when plants were infected with *M. incognita* after 45 days, whereas through single inoculation with *R. variabile* the leaf area was increased and also when AMF and nematodes were co-inoculated (0 days). Plants inoculated simultaneously (0 days) with AMF and *M. incognita* had similar weights as the non-mycorrhizal control; meanwhile, plants infected with nematodes 45 days after AMF inoculation increased their weights compared to simultaneous inoculation (0 days) and the non-inoculated control (Table 4). 

**Figure 4:**
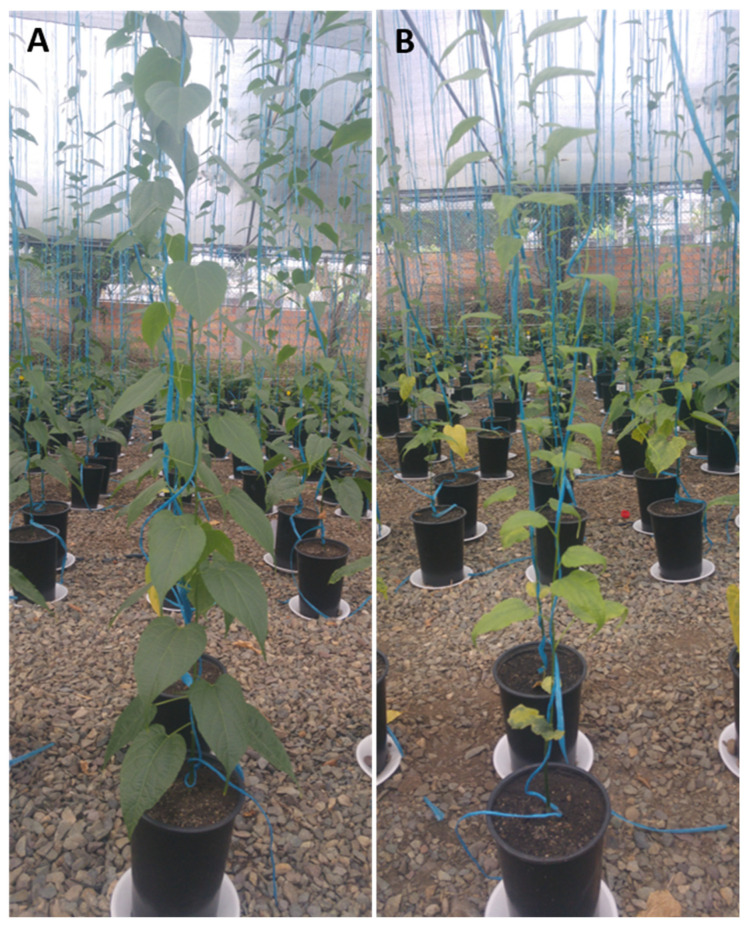
Growth of *Plukenetia volubilis* plants after 75 days. (**A**) After dual inoculation with *Rhizoglomus variabile and Nanoglomus plukenetiae* at planting; (**B**) after infection with *M. incognita* at planting. This figure illustrates chlorophyll content and possibly leaf size.

Chlorophyll content was significantly affected by *M. incognita* infection (0 and 45 days) in the absence of AMF (Table 4; Figure 4) and by the interaction of both factors. Additionally, AMF inoculation and its interaction with *M. incognita* significantly influenced spore density; thus, the highest spore density was generated following dual AMF inoculation in plants infected with *M. incognita* infection at 45 days, but when AMF and nematodes were co-inoculated, *R. variabile* produced the largest number of spores (Table 4), and without significant differences to *R. variabile* inoculated at day 0 together with the nematode infestation. Finally, AMF inoculation significantly influenced mycorrhizal colonization, while *M. incognita* and the interaction of both factors were non-significant (Table 4). Plants inoculated with *R. variabile* presented the highest level of colonization, followed by dual inoculation and single inoculation with *N. plukenetiae*, regardless of the timing of *M. incognita* inoculation but with relatively similar colonization levels, as observed in Experiment #1 for plants without nematode infection.

**Figure 5:**
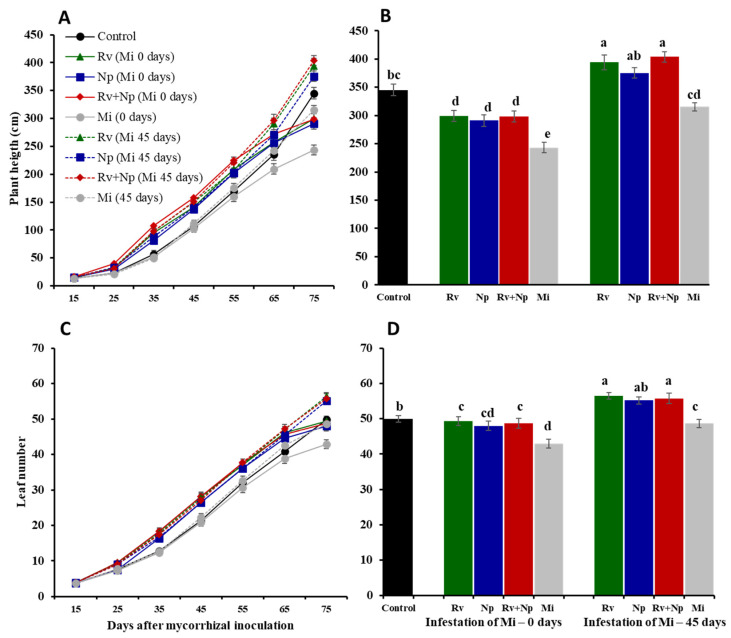
Effects of single and dual inoculation of the arbuscular mycorrhizal fungi *Rhizoglomus variabile* (Rv) and *Nanoglomus plukenetiae* (Np) and infection with *Meloidogyne incognita*. (**A**,**C**): plant height and leaf number of *Plukenetia volubilis* measured at 10-day intervals and (**B**,**D**) at 75 days after mycorrhizal inoculation. Error bars indicate standard deviation (± S.D.). Treatments sharing the same letter are not significantly different (*p* < 0.05). Control = non-inoculated, Mi = infestation with *M. incognita* at 0 and 45 days, Rv + (Mi0 or Mi45) = inoculation with *R. variabile* and infestation with *M. inocognita* at 0 or 45 days, Np + (Mi0 or Mi45) = inoculation with *N. plukenetiae* and infestation with *M. incognita* at 0 or 45 days, Rv + Np (Mi0 or Mi45) = inoculation with *R. variabile* + *N. plukenetiae* and infestation with *M. inocognita* at 0 or 45 days.

Plant height and number of leaves followed a similar trend, i.e., growth was improved when plants were infected by nematodes at 45 days after AMF inoculation (up to 1.17 times more than the control, respectively), whereas when co-inoculated at day 0, growth parameters were more similar to the non-mycorrhizal control but still significantly increased when compared to plants infected with *M. incognita* without AMF inoculation (Figure 5).

#### 3.2.2. Effects of AMF Inoculation on *Meloidogyne incognita*

**Figure 6:**
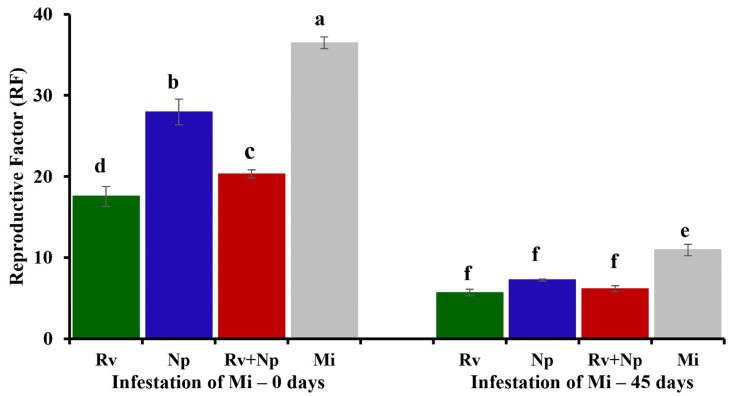
Reproductive factor of *Meloidogyne incognita* assessed in *Plukenetia volubilis* plants inoculated with single and dual inoculation of *Rhizoglomus variabile* (Rv) and *Nanoglomus plukenetiae* (Np). Treatments sharing the same letter are not significantly different (*p* < 0.05). Mi = infestation with *M. incognita* at 0 and 45 days, Rv + (Mi0 or Mi45) = inoculation with *R. variabile* and infestation with *M. incognita* at 0 or 45 days, Np + (Mi0 or Mi45) = inoculation with *N. plukenetiae* and infestation with *M. incognita* at 0 or 45 days, Rv + Np (Mi0 or Mi45) = inoculation with *R. variabile* + *N. plukenetiae* and infestation with *M. incognita* at 0 or 45 days.

Although nematode population densities increased in all treatments, the density of nematodes in roots and soil was significantly influenced by AMF inoculation, *M. incognita* infestation, and the interaction of both factors. The single inoculation with *R. variabile* reduced nematode multiplication the most, with population densities up to 9.8 and 2.3 times lower in roots and soil, respectively, compared with non-mycorrhizal control plants (Table 5). The reproductive factor of *M. incognita* was also significantly affected by AMF inoculation, *M. incognita* infestation, and the interaction of both factors. Inoculation with *R. variabile*, both alone and in combination with *N. plukenetiae*, proved to be more effective in significantly reducing the reproductive factor of *M. incognita*, both at 0 and 45 days of infestation (Rv = −51.87%, Rv + Np = −44.23%, 0 days; Rv = −47.92%, Rv + Np= −43.61%, 45 days). Infestation with *M. incognita* at 45 days resulted in lower reproductive factors in all treatments compared to infestation at 0 days, once again highlighting the effectiveness of the *R. variabile* (Figure 6). The level of nematode infection in roots was lower in plants inoculated with AMF, especially when nematodes were inoculated at 45 days.

#### 3.2.3. Leaf Nutrient Levels 

**Figure 7:**
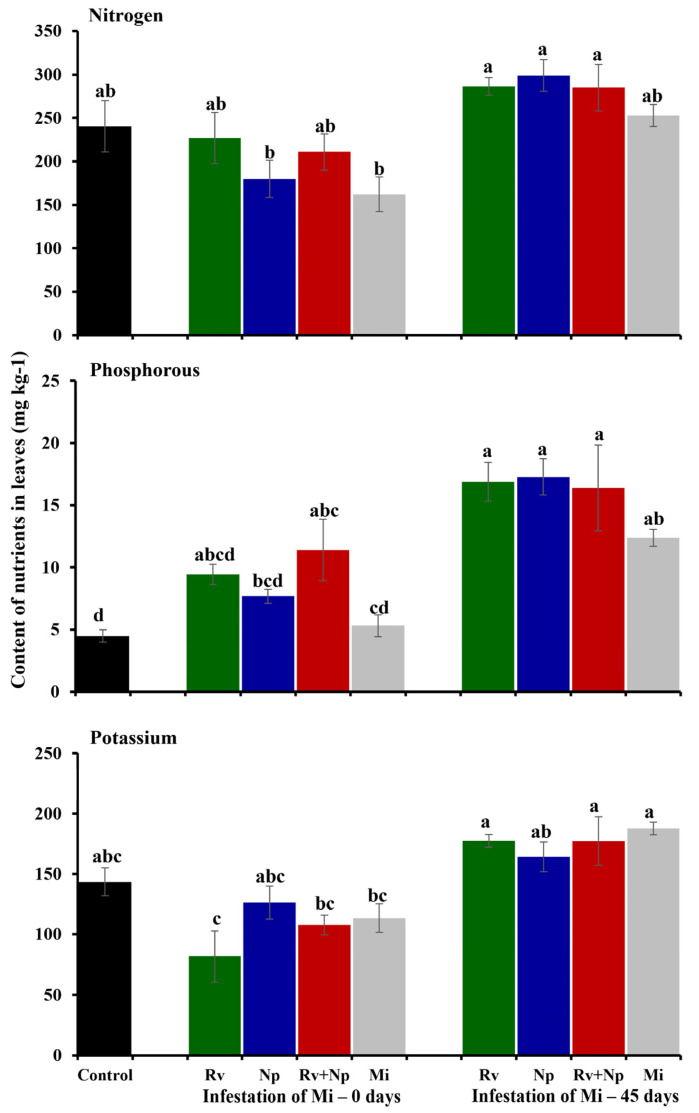
Effect of single and dual inoculation of the arbuscular mycorrhizal fungi *Rhizoglomus variabile* (Rv) and *Nanoglomus plukenetiae* (Np) and the pathogenic nematode *Meloidogyne incognita* on nitrogen, phosphorus, and potassium contents (mg kg^-1^) of *Plukenetia volubilis* leaves after 75 days. Error bars indicate standard deviation (± S.D.). Columns with the same letter are not significantly different (*p* < 0.05).

The phosphorus contents in the leaves were influenced by *M. incognita* infection (0 and 45 days). No effects on N contents were revealed when compared to the non-mycorrhizal controls. AMF inoculation had no significant effect on this variable, either alone or in interaction with nematode infestation. Phosphorus significantly increased in plants inoculated with AMF and infested with *M. incognita* at 45 days compared to plants co-inoculated with AMF and *M. incognita* at 0 days (up to 1.5 and 1.3 times, respectively) and the non-mycorrhizal control (up to 3.9 and 1.2 times, respectively). Similarly, potassium content was significantly influenced by the time of *M. incognita* infection (0 or 45 days) and its interaction with AMF (nematodes: F_1_ = 37.84, *p* < 0.0001; AMF × nematodes: F_3_ = 3.60, *p* = 0.03). In this tendency (and up to 1.40 times), potassium contents increased in plants inoculated with AMF and infested with *M. incognita* at 45 days compared to plants co-inoculated with AMF and *M. incognita* at 0 days and the non-mycorrhizal control (up to 1.24 times) (Figure 7).

## 4. Discussion

The potential for exploiting AMF as a bio-input to improve nutrient use efficiency and, consequently, crop yields has been a long-standing source of attention [31]. Commercially marketed products are now available internationally, which tend to rely on a small number of species/isolates that have proved easier to bulk [32] but may not necessarily be better adapted to the prevailing conditions of the user. In Amazonia, the use of AMF is similarly attracting increasing interest for its use in various economically important crops [33,34,35]. However, despite a small number of studies to assess the impact of AMF with *P. volubilis* [14,15], our study appears to be the first to evaluate the use of native AMF. We assessed the two species *R. variabile* and *N. plukenetiae*, which were originally described from the rhizosphere of *P. volubilis* in western Amazonia [16,17]. The importance of using native species, which are physiologically and genetically adapted locally, has previously been highlighted, as they can promote greater benefits compared to commercially produced fungi derived from different environmental conditions [36,37]. In our study, compelling benefits from using *R. variabile* were evident. Improved fitness of plants inoculated with AMF was clearly observed, which was further pronounced in the presence of root knot nematodes. Indeed, inoculation of *P. volubilis* seedlings prior to exposure to *M. incognita* provided high levels of protection. Although this did not provide total protection, with *M. incognita* still able to multiply, inoculation of *P. volubilis* led to much-reduced nematode infection of roots, up to ~10-fold, even. This translates into a highly recommendable practice for improving *P. volubilis* production. The current study was conducted under controlled conditions in pots, and for a more practical understanding of how this would protect and impact crop production for farmers, field studies would be necessary. Inoculation of seedlings in the nursery prior to transplanting would lead to improved production due to more efficient nutrient acquisition, including through better-protected and thus healthier, more efficient root systems. As root knot nematodes are intractable, difficult-to-control pests and a serious threat to *P. volubilis* [13], the use of native AMF, especially *R. variabile*, provides a tangible alternative management option. However, it remains important to consider the activity of native AMF in the context of varying conditions and soil or agricultural practices [38,39]. Consequently, further assessment is necessary to ensure compatibility of the selected AMF across a range of conditions and, indeed, in combination with other AMF species. 

In the combined application, our study echoed results from other studies, which demonstrated a general lack of synergism between AMF strains [18,40], although with some additional benefits experienced from the dual inoculation, as also observed elsewhere [15]. In general, the total number of associated AMF species may not necessarily be a good predictor of overall benefits provided by AMF, and other aspects, such as the phylogenetic relatedness between species, should also be considered [41]. For example, the three globally most prevalent and commercially used AMF species (*Rhizoglomus intraradices*, *Funneliformis mosseae,* and *R. irregulare*) all belong to the Glomeraceae family [42] and should probably not be combined but instead complemented with species from other AMF families. AMF families have complementary functional capacities in favor of the plant hosts and, as such, AMF species combinations should ideally be comprised of species from different families [43,44]. Combining species with mutually beneficial properties is currently viewed as a step towards developing the so-called “next generation” of inoculant biostimulant products [45] and towards delivering products with multiple benefits in a complementary and synergistic manner.

In our assays, AMF-treated plants were taller and had a greater leaf area compared to non-inoculated controls. Our results are consistent with growth benefits observed in other species from the Euphorbiaceae family, such as *Mallotus paniculatus* using *Gigaspora decipiens* [46] and *Euphorbia pulcherrima* employing a mixture inoculum of *Claroideoglomus claroideum* (currently *Entrophospora claroidea* according to Błaszkowskia et al. [47]), *Rhizoglomus intraradices, Funnelifomis mosseae,* and *F. geosporus* [48]. The plant leaf nutrient contents are also a strong indicator of plant nutrition and health status [49], and our results revealed significantly higher leaf P contents in AMF-treated *P. volubilis*, especially by *N. plukenetiae*, and P and K contents in the dual inoculated plants. Enhanced nutrient uptake by AMF-colonized plants occurs due to an extended root surface area through the hyphal network [50]. In the case of P, however, improved absorption is also achieved through mineralization of organic P by way of hyphal phosphatase exudate and solubilization of inorganic P via organic acid production and pH modification of the rhizosphere [51], as well as the induction of the host phosphate transporter gene expressed on the peri-arbuscular membrane [52]. Various studies have consistently demonstrated the improvements in P acquisition by using AMF inoculation in various Euphorbiaceae species [53] and other botanical families [54]. An increase of up to 2.5-fold in P content was recorded in our study, which is high compared with other studies, such as 1.5-fold in *Manihot esculenta* following inoculation with *Acaulospora colombiana* and *Ambispora appendicula* [55]. The difference in leaf P content of AMF-inoculated plants was further increased, however, when challenged with *M. incognita*, with up to 3.9-fold higher P content than non-AMF plants compared with other studies, such as in *Carica papaya* with *R. irregulare* [56] and in *Solanum lycopersicum* with *F. mosseae* [57], which showed 1.3 times higher P contents. The raised K leaf content in the dual AMF inoculation treatment only may be the result of enhanced P uptake, as a strong association between K and P during AMF symbiosis has been described [58,59]. Despite our expectations, however, no increase in N content was recorded following AMF inoculation. This reflects the results of Reynolds et al. (2005), which established that mycorrhizal symbiosis could have a neutral or negative effect on host N intake. This situation may be explained by the “trade balance model” [60], which suggests that the increase in N uptake will only occur if the plant is restricted by P and hence will benefit from providing carbon (C) to the roots and associated mycorrhizal fungi. 

With respect to the protective effect of AMF against biotic stress factors [7], there are numerous accounts of AMF enhancing resistance against plant pathogens by competing for colonization sites and improving plant defense systems [61]. For plant parasitic nematodes, most attention has generally tended to focus on protection against root knot nematodes (*Meloidogyne* spp.), with repeated demonstrations of AMF application suppressing nematode reproduction and root galling damage [62]. However, this interaction also appears complex and can be dependent on various factors, such as AMF, plant, or nematode species, as well as environmental conditions [63]. In our study, AMF provided substantial protection against *M. incognita*. Application of AMF, therefore, would undoubtedly be beneficial in the management of the soil-borne complex (*M. incognita* and *Fusarium* spp.) that can be devastating to *P. volubilis* [13] and should be further assessed. The application of AMF in the nursery prior to transplanting led to higher levels of protection, especially by *R. variabile*. It is likely that early root colonization affords AMF the opportunity to compete for space against nematodes, which promotes structural and morphological alterations in the root system to counterbalance infection, in addition to providing an enhanced nutritional host plant state [62,64] and activating plant defense priming [65]. 

For an AMF species or isolate that demonstrates benefits and advantages for their hosts to be successfully developed into a product, the isolate must also possess additional qualities or traits, such as durability, high multiplication factor, colonization, etc. [66,67,68]. Species of *Rhizoglomus* are characterized by their ability to produce abundant spores [69], while their beneficial influence on plant growth has been demonstrated on various hosts, such as Phoenix dactylifera [70], *Abelmoschus esculentus* [71], and *Robinia pseudoacacia* [72]. The genus *Rhizoglomus*, along with *Nanoglomus*, belong to the Glomeraceae family, which are broadly known for their early colonization, high rates of growth, and short generation times [66]. In our study, *R. variabile* exhibited optimal values of spore density and root colonization and suppressed *M. incognita* reproduction, particularly in plants inoculated with *M. incognita* after 45 days of inoculation with AMF.

## 5. Conclusions

This study demonstrates the biofertilizer and bioprotective impact of two species of AMF native to Amazonia on the indigenous crop *P. volubilis*. Both *R. variabile* and *N. plukenetiae* improved early growth and development of *P. volubilis* and, importantly, provided a high level of protection against the root knot nematode, *M. incognita*. However, better results in plant fitness occurred when the plant was inoculated with *R. variabile*. These findings are a first step towards advocating for the use of AMF as a mechanism for improving the sustainable production of this crop.

## Figures and Tables

**Table 1:** Summary of Experiment #1 treatments to evaluate the effects of AMF inoculation on *Plukenetia volubilis*.

Treatment	Description
Control	Non-inoculated
Rv	Inoculation with *Rhizoglomus variabile*
Np	Inoculation with *Nanoglomus plukenetiae*
Rv + Np	Inoculation with *Rhizoglomus variabile* + *Nanoglomus plukenetiae*

**Table 2:** Summary of Experiment #2 treatments to evaluate AMF against *Meloidogyne incognita* in *Plukenetia volubilis*.

Treatment	Description
Control	Non-inoculated
Rv/Mi 0	Inoculation with *Rhizoglomus variabile* and *Meloidogyne incognita* at 0 days
Np/Mi 0	Inoculation with *Nanoglomus plukenetiae* and *Meloidogyne incognita* at 0 days
Rv + Np/Mi 0	Inoculation with *R. variabile* + *N. plukenetiae* and *M. incognita* at 0 days
Mi 0	Infestation with *Meloidogyne incognita* at 0 days
Rv/Mi 45	Inoculation with *Rhizoglomus variabile* and *Meloidogyne incognita* at 45 days
Np/Mi 45	Inoculation with *Nanoglomus plukenetiae* and *Meloidogyne incognita* at 45 days
Rv + Np/Mi 45	Inoculation with *R. variabile* + *N. plukenetiae* and *M. incognita* at 45 days
Mi 45	Infestation with *Meloidogyne incognita* at 45 days

**Table 3:** Impact of AMF inoculation on *Plukenetia volubilis* growth, colonization, and physiology after 75 days.

Treatment ^1^	Leaf Area (cm^2^)	Chlorophyll Content (SPAD)	Total Fresh Biomass (g)	Shoot Dry Biomass (g)	Root Colonization (%)	Spore Density (10 g Soil)
Control	493 ± 11.5 c	35.7 ± 0.48 a	77.9 ± 2.5 b	10.6 ± 0.32 b	0	0
Rv	652 ± 8.3 a	38.5 ± 0.67 a	90.4 ± 2.0 a	14.4 ± 0.24 a	94.4 ± 1.0 a	169 ± 5.2 a
Np	609 ± 8.1 b	36.9 ± 0.94 a	88.7 ± 1.3 a	13.8 ± 0.26 a	73.1 ± 1.3 c	74 ± 6.7 c
Rv + Np	613 ± 5.4 b	37.6 ± 1.03 a	89.5 ± 1.4 a	13.8 ± 0.39 a	90.1 ± 1.1 b	114 ± 6.1 b
*p*-values	*p* < 0.0001	*p* = 0.1129	*p* < 0.0001	*p* < 0.0001	*p* < 0.0001	*p* < 0.0001
F-values	F_3_ = 63.792	F_3_ = 2.110	F_3_ = 10.129	F_3_ = 30.567	F_2_ = 98.287	F_2_ = 62.252

^1^ Rv = *Rhizoglomus variabile* and Np = *Nanoglomus plukenetiae.* Means ± standard deviation of 12 replicates. Treatments with the same letter are not significantly different within a column (*p* < 0.05).

**Table 4:** Impact of AMF single and dual inoculation and nematode infestation on *Plukenetia volubilis* growth and physiology after 75 days.

Treatment ^1^	Leaf Area (cm^2^)	Chlorophyll Content (SPAD)	Total Fresh Biomass (g)	Shoot Dry Biomass (g)	Root Colonization (%)	Spore Density (10 g Soil)
Control	493 ± 12 de	35. 7 ± 0.5 a	77.9 ± 2.5 b	10.6 ± 0.32 b	0	0
Co-inoculation of AMF and *M. incognita* at 0 days						
Rv/Mi 0	563 ± 9 bc	38.3 ± 0.6 a	79.9 ± 0.8 b	10.5 ± 0.31 b	93.2 ± 0.6 a	101 ± 12.6 ab
Np/Mi 0	522 ± 7 cd	36.3 ± 0.6 a	81.2 ± 1.6 b	9.5 ± 0.39 b	71.1 ± 0.9 c	41 ± 4.0 c
Rv + Np/Mi 0	539 ± 11 cd	36.6 ± 0.8 a	80.9 ± 1.3 b	9.4 ± 0.24 b	88.5 ± 1.3 b	65 ± 5.9 bc
Mi 0	423 ± 16 f	28.3 ± 1.1 c	57.1 ± 3.6 c	5.6 ± 0.29 c	0	0
Inoculation of *M. incognita* after 45 days						
Rv/Mi 45	630 ± 9 a	36.8 ± 0.6 a	90.9 ± 1.3 a	14.3 ± 0.27 a	93.0 ± 0.7 a	89 ± 2.0 ab
Np/Mi 45	611 ± 10 ab	36.8 ± 0.7 a	89.9 ± 1.8 a	13.4 ± 0.33 a	72.7 ± 0.8 c	46 ± 2.0 c
Rv + Np/Mi 45	637 ± 18 a	36.0 ± 0.6 a	93.6 ± 1.1 a	13.7 ± 0.35 a	91.8 ± 0.5 ab	113 ± 20 a
Mi 45	461 ± 12 ef	32.4 ± 0.8 b	75.4 ± 2.4 b	10.3 ± 0.44 b	0	0
*p* and F-value						
AMF	*p* < 0.0001 F_3_ = 70.809	*p* < 0.0001 F_3_ = 27.713	*p* < 0.0001 F_3_ = 40.662	*p* < 0.0001 F_3_ = 52.178	*p* < 0.0001 F_2_ = 286.970	*p* < 0.0001 F_2_ = 26.84
Nematodes	*p* < 0.0001 F_1_ = 43.742	*p* < 0.0001 F_1_ = 18.755	*p* = <0.0001 F_1_ = 49.466	*p* < 0.0001 F_1_ = 177.729	*p* = 0.034 F_1_ = 4.726	*p* = 0.047 F_1_ = 4.890
AMF × Nematodes	*p* = 0.070 F_3_ = 2.435	*p* = 0.001 F_3_ = 5.838	*p* = 0.116 F_3_ = 2.021	*p* = 0.288 F_3_ = 1.2741	*p* = 0.161 F_2_ = 1.883	*p* = 0.005 F_2_ = 8.188

^1^ Rv = *Rhizoglomus variabile* and Np = *Nanoglomus plukenetiae*. Treatments with the same letter in the column are not significantly different (*p* < 0.05). N = 12 replicate pots.

**Table 5:** Impact of single and dual inoculation of *Rhizoglomus variabile* and *Nanoglomus plukenetiae* AMF on population density of *Meloidogyne incognita* (J2) after 75 days. Rv = *Rhizoglomus variabile* and Np = *Nanoglomus plukenetiae*. Treatments with the same letter are not significantly different within a column (*p* < 0.05). N = 12 replicated pots.

	Nematode Density	
	Per Plant	Per 100 g of Soil
Control	0	0
Co-inoculation of AMF and *M. incognita* at 0 days		
Rv/Mi 0	15319 ± 563.9 c	1244 ± 72.9 c
Np/Mi 0	17860 ± 121.0 b	2200 ± 88.2 b
Rv + Np/Mi 0	16972 ± 279.0 bc	1467 ± 19.3 c
Mi 0	23403 ± 1111.3 a	2867 ± 19.3 a
Inoculation of *M. incognita* after 45 days		
Rv/Mi 45	448 ± 79.0 f	556 ± 22.2 e
Np/Mi 45	1443.8 ± 279.0 e	678 ± 19.3 e
Rv + Np/Mi 45	1194 ± 77.4 e	578 ± 22.2 e
Mi 45	4194 ± 108.5 d	956 ± 40.1 d
*p-* and F-values		
AMF	*p* < 0.0001	*p* < 0.0001
	F_3_ = 119.7	F_3_ = 202.65
Nematodes	*p* < 0.0001	*p* < 0.0001
	F_1_ = 4602	F_1_ = 1517.92
AMF × Nematodes	*p =* 0.018	*p* < 0.0001

## Data Availability

The dataset and associated R codes used in the main results are available upon reasonable request to the corresponding author.
